# SARS-CoV-2 Infection-Induced Promoter Hypomethylation as an Epigenetic Modulator of Heat Shock Protein A1L (HSPA1L) Gene

**DOI:** 10.3389/fgene.2021.622271

**Published:** 2021-02-19

**Authors:** Jibran Sualeh Muhammad, Narjes Saheb Sharif-Askari, Zheng-Guo Cui, Mawieh Hamad, Rabih Halwani

**Affiliations:** ^1^Department of Basic Medical Sciences, College of Medicine, University of Sharjah, Sharjah, United Arab Emirates; ^2^Sharjah Institute for Medical Research, University of Sharjah, Sharjah, United Arab Emirates; ^3^Department of Environmental Health, University of Fukui School of Medical Science, University of Fukui, Fukui, Japan; ^4^Department of Medical Laboratory Sciences, College of Health Sciences, University of Sharjah, Sharjah, United Arab Emirates; ^5^Department of Clinical Sciences, College of Medicine, University of Sharjah, Sharjah, United Arab Emirates; ^6^Prince Abdullah Ben Khaled Celiac Disease Research Chair, Department of Pediatrics, Faculty of Medicine, King Saud University, Riyadh, Saudi Arabia

**Keywords:** SARS-CoV-2, epigenetic, COVID-19, DNA methylation, bioinformatics, HSPA1L

## Abstract

Numerous researches have focused on the genetic variations affecting SARS-CoV-2 infection, whereas the epigenetic effects are inadequately described. In this report, for the first time, we have identified potential candidate genes that might be regulated *via* SARS-CoV-2 induced DNA methylation changes in COVID-19 infection. At first, *in silico* transcriptomic data of COVID-19 lung autopsies were used to identify the top differentially expressed genes containing CpG Islands in their promoter region. Similar gene regulations were also observed in an *in vitro* model of SARS-CoV-2 infected lung epithelial cells (NHBE and A549). SARS-CoV-2 infection significantly decreased the levels of DNA methyltransferases (DNMT1, DNMT3A, and DNMT3B) in lung epithelial cells. Out of 14 candidate genes identified, the expression of 12 genes was upregulated suggesting promoter hypomethylation, while only two genes were downregulated suggesting promoter hypermethylation in COVID-19. Among those 12 upregulated genes, only *HSPA1L* and *ULBP2* were found to be upregulated in AZA-treated lung epithelial cells and immune cells, suggesting their epigenetic regulation. To confirm the hypomethylation of these two genes during SARS-CoV-2 infection, their promoter methylation and mRNA expression levels were determined in the genomic DNA/RNA obtained from whole blood samples of asymptomatic, severe COVID-19 patients and equally matched healthy controls. The methylation level of *HSPA1L* was significantly decreased and the mRNA expression was increased in both asymptomatic and severe COVID-19 blood samples suggesting its epigenetic regulation by SARS-CoV-2 infection. Functionally, *HSPA1L* is known to facilitate host viral replication and has been proposed as a potential target for antiviral prophylaxis and treatment.

## Introduction

In the last 20 years, an upsurge of several novel pathogenic viral infection outbreaks were identified and reported (de Groot et al., 2013; Weber et al., 2016). In December 2019, many serious and unexplained cases with pneumonia-like signs and symptoms became evident in Wuhan, China. The causative viral agent behind these cases was identified to be Severe Acute Respiratory Syndrome Coronavirus-2 (SARS-CoV-2) (Zhou et al., 2020). Later, this novel Coronavirus spread all over the world forming the first coronavirus pandemic (World Health Organization [WHO], 2020; Peiris et al., 2003; Guan et al., 2020; Guo et al., 2020; Sun et al., 2020).

Old age, comorbidities, and male sex are associated with more severe COVID-19 outcomes (Gebhard et al., 2020). Still, many young and healthy individuals have also died of COVID-19, leaving researchers in an enigma. Hence, suggesting a possibility that epigenetic regulation of host factors might play an important role in defining the landscape of COVID-19 disease progression. Epigenetics refers to heritable and acquired alterations in gene activity and expression, without changes in the DNA sequence; which can occur in fully differentiated, post-mitotic cells in response to environmental signals (Jaenisch and Bird, 2003). Epigenetic mechanisms such as DNA methylation and histone modifications have been heavily implicated in the development of cancer, in which silencing of tumor suppressor genes facilitates carcinogenesis (Muhammad et al., 2017, 2018, 2020). Also, several reports have shown that persistent viral infection (e.g., HBV, KSHV, EBV, and HIV) can epigenetically influence host gene expression and might play a major role in the pathogenesis of disease progression (Paschos and Allday, 2010). Interestingly, the cell entry receptor for SAR-CoV-2, ACE2, is transcriptionally regulated by DNA methylation (Tipnis et al., 2000). In fact, this receptor is located on the X chromosome (Tipnis et al., 2000) which may explain the gender differences in susceptibility and progression of COVID-19 (Pruimboom, 2020). Thus far, no study has examined the complete epigenetic landscape of differentially expressed genes following COVID-19 infection.

A high number of epigenetic biomarker candidates are continuously being proposed. Epi proColon^®^ 2.0 CE (Epigenomics AG, Berlin, Germany) is a blood-based test that uses a real-time PCR system and MethyLight assay detects methylated genes to identify colorectal carcinoma patients (Lamb and Dhillon, 2017). In this study, for the first time, we report novel genes that are probably the candidate genes likely to be epigenetically regulated by SARS-CoV-2 infection in lung epithelial and infiltrated immune cells, and those genes might have a role in COVID-19 pathogenesis. These genes could also be used as potential epigenetic biomarkers to rapidly identify SARS-CoV-2 infected asymptomatic individuals and might also help in the stratification of COVID-19 patients. SARS-CoV-2 regulated epigenetic modifications could also be associated with enhanced severity of COVID-19 infection, hence, could be utilized as a potential target for anti-SARS-CoV-2 prophylaxis and treatment.

## Materials and Methods

### Transcriptomic Data Resources for COVID-19 Human Lung Samples

We searched for transcriptomic datasets from National Center for Biotechnology Information Gene Expression Omnibus (GEO)^1^, which is a free public functional genomics database including array- and sequence-based data. The search terms COVID-19 and Lungs were used to identify datasets for human lungs infected with COVID-19. We selected dataset GSE150316, which is a high throughput sequencing dataset of autopsy samples from each of the five different patients deceased due to SARS-CoV-2 infection. In this dataset, a rapid autopsy was performed for sample collection and samples were formalin-fixed paraffin-embedded before the RNA extraction was done. At least two and a maximum of five tissue sections from areas devoid of acute inflammation were used per sample and a total of five healthy control lung samples were utilized for comparison^2^. For analysis in our study, all the technical replicates for each SARS-CoV-2 infected and negative control samples were averaged for the downstream analysis. The differential expression analysis was performed using the DeSeq2 function to call out the differentially expressed genes (DEGs) comparing mean expression from each patients’ technical replicates vs. the mean expression of negative control samples. The fold change from these five pairwise comparisons was integrated by Venn analysis and presented as Venn diagram. Later, the fold change expression obtained from each of the pairwise comparisons was grouped and averaged and log2fold change was calculated and presented in a bar graph as relative mRNA expression of infected vs. control.

### Transcriptomic Data Resources for Chronic Inflammatory Human Lung Samples

Next, we sought to compare the COVID-19 gene expression profile with a chronic inflammatory lung condition. Hence, the search terms COPD and Lungs were used to identify datasets for the human lungs of chronic obstructive pulmonary diseases (COPD). We selected dataset GSE57148, in which the authors have performed gene expression profiling of lung tissue obtained from 98 COPD subjects and 91 normal subjects (Jeong et al., 2018). For analysis in our study, all the COPD subjects and normal subjects were averaged and the DEGs were called out comparing mean expression from COPD patients vs. the mean expression of the normal subjects. Log2fold change was calculated as mean of relative mRNA expression of all the five COVID-19 samples vs. negative control and were presented in a bar graph alongside the COVID-19 sample data, both as a separate analysis.

### Identification of CpG Islands in the Promoter Region of DEGs

All the ubiquitously expressed genes in COVID-19 lung tissue were searched through the NCBI database using GRCh38/hg38 assembly (Genome Reference Consortium) to identify the presence of CpG Island (CGI) in their promoter region. As described previously (Muhammad et al., 2020), Genes were filtered based on the presence of CGI upstream of transcription start site with GC content of >50%, length > 200 bp and ratio > 0.6 of observed/expected number of CG dinucleotides and based on the number of Gs and Cs in the DNA segment. A total of 19 genes were selected, which are highly suspected to be epigenetically regulated by hyper/hypo-methylation of their promoter region.

### Analysis of Cell-Specificity of DEGs in Human Lung

Lung Gene Expression Analysis Web Portal^3^ was utilized to identify the selective expression of our candidate genes across different cell types in human lung tissue. This online platform contains a dataset of RNAseq analysis of 81 samples derived from 12 LungMAP donor subjects ranging in age from 1 day of life to adult. Selective gene expression in each cell type is presented as a pie chart.

### Expression Profiling in Normal Human Tissues

To have a better understanding of the expression profile of all the DEGs in COVID-19 infection, the Genotype-Tissue Expression (GTEx) online portal^4^ was used. This tool is a comprehensive public resource to study tissue-specific expression levels and regulation comprising of samples collected from 54 non-diseased tissue sites across nearly 1,000 individuals from a publicly available database. The candidate DEGs identified from COVID-19 infected lung tissues dataset and are most likely to be epigenetically regulated were searched in this database and are presented as expression heat maps across all the tissues and its expression profile in normal lungs.

### Transcriptomic Data Resources for Human Cell Lines

Again, the NCBI GEO database was searched to identify relevant studies showing the effect of SARS-CoV-2 infection-induced expression of the candidate genes. Dataset GSE147507 containing expression profiling by high throughput sequencing from independent biological triplicates of primary human lung epithelium (NHBE) cells and transformed normal lung alveolar (A549) cells mock-treated or infected with SARS-CoV-2 were selected for reanalysis and differential gene expression of mock-treated vs. SARS-CoV-2 treated cells was searched for our list of candidate genes (Blanco-Melo et al., 2020).

Datasets GSE32496 and GSE18454 were selected for analyzing the effect of DNA demethylation on the expression of those candidate genes. These datasets were containing expression profiling by array from NHBE (GSE18454) and A549 (GSE32496) cells untreated or following treatment with 5-aza-dC (AZA; 0.5 μM) (Heller et al., 2013; Blanco-Melo et al., 2020).

### Quantitative Methylation-Specific PCR

Total genomic (g)DNA was extracted from whole blood samples obtained from the healthy controls (*n* = 5) and COVID-19 patients (*n* = 5 for each asymptomatic and symptomatic) using QIAamp DNA Mini Kit mini kit (Qiagen, Hilden, Germany).

An aliquot gDNA (2 μg) was treated with EpiTect Bisulfite Kit (Qiagen). qMSP was conducted using 1 μl of the sodium bisulfite-treated DNA, primers specifically designed for methylated and unmethylated DNA sequence of the promoter region of *HSPA1L* and *ULBP2* genes (Supplementary Table 1), Promega GoTaq^®^ qPCR Master Mix (Promega); and analyzed using Qiagen Rotor-gene qPCR machine (Qiagen). Based on the (cycle threshold) Ct values of methylated (M) primer amplification and un-methylated (U) primer amplification for a gene, a methylation level of the gene promoter was calculated as the fraction of M molecules in the total number of bisulfite-treated DNA molecules (Ct of M molecules/Ct of M molecules + Ct of U molecules) (Paschos and Allday, 2010). For each gene, methylation levels of asymptomatic COVID-19, as well as symptomatic COVID-19 were compared amongst healthy control. Fully methylated and fully unmethylated control DNAs were purchased from Qiagen and were used as positive and negative controls, respectively and to assess the behavior and specificity of the primers used in this study (Supplementary Figure 1).

### RNA Extraction and Quantitative Real-Time Reverse Transcription (RT)-PCR

Total RNA was extracted from whole blood samples obtained from the healthy controls (*n* = 5) and COVID-19 patients (*n* = 5 for each asymptomatic and symptomatic) using the Trizol-based method. Whole blood clots were added into 750 μl of Trizol^®^ LS reagent (Invitrogen, CA, United States). Mixtures were then gently inverted 5–8 times and incubated at room temperature for 15 min. After which, 200 μl of chloroform (J.T. Baker, PA, United States) was added. Samples were mixed by inverting for 15 s and once more incubated at room temperature for 5 min. After centrifugation at 12,000 *g* for 15 min, at 4°C, the upper aqueous phase was carefully transferred into a new tube, upon which 500 μl isopropanol (J.T. Baker, PA, United States) was added, and then incubated for 10 min at room temperature, before then being centrifuged at 4°C, 12,000 *g* for 10 min. Pellets were washed with 75% ethanol (J.T. Baker, PA, United States), air-dried at room temperature for 30 min, and re-suspended in 20 μl of RNase-free water (Qiagen, Hilden, Germany). Concentration and purity of the total RNA were assessed by NanoDropND-2000 Spectrophotometer (Thermo Scientific, MA, United States) at an optical density (OD) ratio of A260/280 and A260/230 nm.

Complementary cDNA was synthesized using the QuantiTect Reverse Transcription Kit (Qiagen) from 1 μg of RNA according to the manufacturer’s protocol. RT-PCR was performed using 1 μl of cDNA, specific primers (Supplementary Table 1), Promega GoTaq^®^ qPCR Master Mix (Promega, Madison, WI, United States) and Qiagen Rotor-gene qPCR machine (Qiagen). Cycling conditions for qRT-PCR were as follows: 95°C for 15 min, followed by 50 cycles of 94°C for 15 s, 60°C for 30 s, and 70°C for 30 s. Experiments were performed in triplicate, with the mean values for Ct values being used for the calculation. Expression levels of target genes were normalized to GAPDH. GAPDH expression Ct value was always within 0.5 cycles if derived from the same cDNA sample, and cDNA samples from different treatment varied only by 1–2 cycles between samples. To ensure accurate normalization on every real-time assay, the “gene of interest” amplification data was normalized with housekeeping data derived from the same cDNA sample.

### Transcriptomic Data Processing and Statistics

The gene expression profiles were downloaded from the GEO database. Raw data from each dataset were processed using R statistical software (version 3.5.1). According to the expression profiling data, DEGs were presented as relative mRNA expression in infected/treated samples compared vs. the normal or negative controls were identified using the Limma package (available at^5^) in Bioconductor package version 1.0.2. A log2 fold-change (log2FC) was calculated using the average expression for all pairwise comparisons to present DEGs and an adjusted *p*-value < 0.05 using classical t-test was applied. For qMSP analysis, the significant difference was estimated by the student’s t-test considering unequal variance and a *p*-value of <0.05 was considered significant. For all the pooled data were presented as mean ± standard error of the mean (SEM).

### Ethics

Dubai Scientific Research Ethics Committee (DSREC) approved the use of human blood samples in this study. Written informed consent was obtained from all study participants before inclusion.

## Results

### Differential Expression of 101 Common Gene Signatures in Lung Autopsies of Five COVID-19 Patients

**FIGURE 1:**
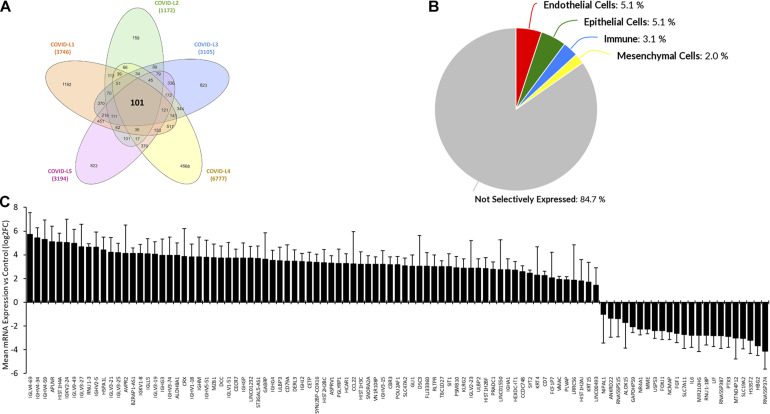
Common differential expressed gene signatures in lung autopsies from five COVID-19 patients. **(A)** RNAseq data set containing expression profile from autopsy samples (Formalin-fixed paraffin-embedded lung tissues) from five patients deceased due to SARS-Cov2 infection, were analyzed. Each patient sample had three or more replicates. Mean expression was calculated and Log2 fold change was computed for each patient sample. Common differentially expressed genes were identified with a threshold of a Log2FC of 1.5 or above. Non-coding genes or pseudogenes were excluded. Total 101 genes were selected based on these criteria and the Venn diagram was constructed to represent the common genes. **(B)** Database containing 81 RNAseq samples derived from 12 Healthy lung donor subjects was utilized to identify selectivity of these commonly expression 101 genes. Most of the genes were non-selectively expressed. **(C)** Bar graph showing expression of those 101 genes in COVID-19 lung tissue compared to normal lung. All the values are representing Log2FC ± SEM for the relative mRNA expression in five COVID-19 cases compared vs. healthy controls.

*In silico* transcriptomic data sets of lung autopsies from five patients who died with COVID19 were used. The data was analyzed to determine the DEGs within these autopsies. Common differentially expressed genes were identified with a threshold of Log2FC of 1.5 and above. A total of 101 genes were selected as COVID-19 lung signatures by taking the intersection of DEGs from all the five patients (Figure 1A). We then used the Lung Gene Expression Analysis portal to identify the selective expression of these 101 genes. Most of the genes were non-selectively expressed (Figure 1B). Further a bar graph was plotted showing the expression of these 101 genes in COVID-19 lung tissue compared to normal lung (Figure 1C).

### Nineteen of the Common Differentially Expressed Genes Had CpG Island in Their Promoter Region

**FIGURE 2:**
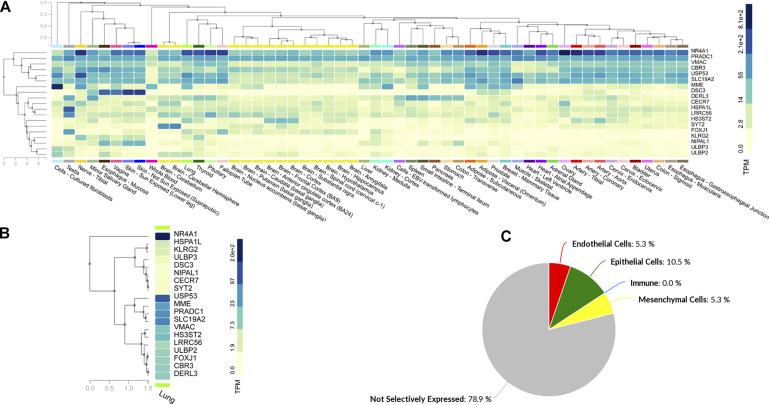
Nineteen of the common differentially expressed genes had CpG Island in their promoter region. **(A)** Data from the Genotype-Tissue Expression (GTEx) database containing normal tissue expression datasets were utilized to analyze the tissue-specific expression levels of all the ubiquitously expressed genes. **(B)** GTEx database was utilized to present the gene expression profile of 19 short-listed epigenetic genes in normal human lung tissue (TPM: transcripts per millions). **(C)** A database containing 81 RNAseq samples derived from 12 Healthy lung donor subjects was utilized to identify cell-specific selectivity of these 19 epigenetic genes. (N.B.: *Endothelial Cells:* ULBP2; *Epithelial Cells:* FOXJ1, LRRC56; *Immune Cells:* None; *Mesenchymal Cells:* MME; *Non-selectively expressed:* CBR3, CECR7, DERL3, DSC3, HS3ST2, HSPA1L, KLRG2, NIPAL1, NR4A1, PRADC1, SLC19A2, SYT2, ULBP3, USP53, and VMAC).

To identify the potential epigenetically modified genes among these 101 common genes, we searched the NCBI database for the presence of CpG Islands in their promoter regions. Out of 101 genes, 19 genes were selected. The names and functions of the shortlisted genes are presented in Table 1. The tissue-specific expression of the 19 genes was assessed using the GTEx database. There was no difference in the abundance of these genes between the lungs and the rest of the body tissues (Figures 2A,B). The top genes that were widely expressed across different organs were *NR4A1*, *USP53*, *MME*, *PRADC1*, and *SLC19A2*. We also used the Lung Gene Expression Analysis portal to identify the selective expression of these 19 genes. Like the 101 DEGs, most of the 19 epigenetic regulated genes were non-selectively expressed. Some of the genes such as the *ULBP2* gene was selectively expressed by endothelial cells, *FOXJ1* and *LRRC56* were only expressed by epithelial cells, and the *MME* gene by mesenchymal cells (Figure 2C).

**TABLE 1:** List and function of the genes containing CpG Island in their promoter region.

**Gene**	**Function (Genecards.com)**
*CBR3*	Catalyzes the reduction of many active carbonyl compounds to their corresponding alcohols
*CECR7*	RNA Gene, and is affiliated with the lncRNA class
*DERL3*	The functional component of endoplasmic reticulum-associated degradation for misfolded luminal glycoproteins
*DSC3*	Component of intercellular desmosome junctions and involved in cell-cell adhesion
*FOXJ1*	Role in cell fate determination during lung development and spermatogenesis
*HS3ST2*	Sulfotransferase that utilizes 3′-phospho-5′-adenylyl sulfate (PAPS) to catalyze the transfer of a sulfo group to an N- unsubstituted glucosamine linked to a 2-O-sulfo iduronic acid unit on heparan sulfate
*HSPA1L*	In cooperation with other chaperones, Hsp70s stabilize preexistent proteins against aggregation and mediate the folding of newly translated polypeptides in the cytosol as well as within organelles
*KLRG2*	An important paralog of this gene is CD69
*LRRC56*	Required for the assembly of dynein arms
*MME*	Neutral endopeptidase that inactivates several peptide hormones
*NIPAL1*	Acts as an Mg^2+^ transporter. Can also transport other divalent cations
*NR4A1*	Orphan nuclear receptor regulates the expression of delayed-early genes during liver regeneration
*PRADC1 (C2ORF7)*	Protease-associated domain-containing glycoprotein
*SLC19A2*	High-affinity transporter for the intake of thiamine
*SYT2*	Regulatory role in the membrane interactions during trafficking of synaptic vesicles at the active zone
*ULBP2*	Encodes MHC class I-related molecule that binds to the NKG2D receptor on NK cells to trigger the release of multiple cytokines and chemokines that in turn contribute to the recruitment and activation of NK cells
*ULBP3*	Ligands of the KLRK1/NKG2D receptor, which is found in primary NK cells
*USP53*	Modulates the barrier properties and mechanical stability of tight junctions
*VMAC*	Vimentin-type intermediate filament associated coiled-coil protein

*All the commonly expressed genes in COVID-19 lung tissue were searched through the NCBI database using GRCh38/hg38 assembly (Genome Reference Consortium) to identify the presence of CpG Island in the promoter region.*

### The Selected Genes Are Upregulated During COVID-19, but Not Due to Chronic Lung Inflammation

**FIGURE 3:**
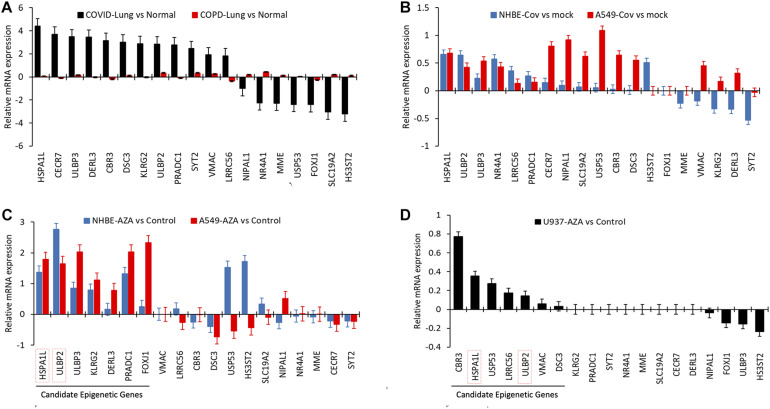
The epigenetic candidate genes are upregulated during COVID-19, but not due to chronic lung inflammation. **(A)** Relative mRNA expression (Log2FC) of 19 selected epigenetic genes in COVID-19 infected lung tissue were plotted and were plotted against Relative mRNA expression of the same genes in lung tissues obtained from COPD patients (distinctive healthy controls RNAseq data were used as a control for each disease-type). **(B)** Relative mRNA expression (FC) of 19 selected epigenetic genes in human lung epithelial cell lines infected with SARS-Cov-2. GEO data set containing expression profiling by high throughput sequencing of human lung epithelial cell lines infected with SARS-Cov-2 was searched. Dataset included had independent biological triplicates of primary human lung epithelium (NHBE) mock-treated or infected with SARS-CoV-2 and independent biological triplicates of transformed lung alveolar (A549) cells mock-treated or infected with SARS-CoV-2. **(C,D)** GEO data set containing expression profiling by high throughput sequencing of human lung epithelial cell lines (NHBE, A549) and monocyte cell line (U937) treated with 0.5 μM Aza-dC (AZA) were analyzed for mean mRNA expression (FC) of these 19 epigenetic genes. Genes that are found upregulated are more likely suspected to be epigenetically regulated in lung epithelial cells or immune cells. All bar graph data represents mean relative mRNA expression for pairwise comparison of infected/treated samples vs. the control and error bars represents SEM.

For comparison against chronic lung inflammatory conditions, the lung expression of these 19 candidate genes in the COVID dataset was plotted the expression dataset from COPD patients. Distinctive healthy controls RNAseq data were used for comparison as a control for each disease-type (Figure 3A). This comparison showed that the expression of those 19 genes was not changed in lung tissues of COPD patients. *ULBP2*, *SYT2*, *VMAC*, and *FOXJ1* followed the same pattern of expression in COPD compared to COVID-19, however, to a much lower level. The opposite pattern, however, was observed for *LRRC56* and *NR4A1* genes. We then evaluated the expression of these 19 genes within datasets of epithelial cells infected, *in vitro*, with SARS-CoV-2 (NHBE and A549). The overall expression of these genes was much lower in epithelial cells compared to lung autopsies. Although we detected a similar overall expression pattern in infected epithelial cells and lung autopsies, there was a notable difference in the pattern of expression of some genes including *NR4A1* and *NIPAL1*; these were downregulated in COVID-19 lung autopsies but upregulated in infected epithelial cells (Figure 3B).

### AZA Treatments of Epithelial and Immune Cells Confirm Epigenetic Regulation of 12 Genes

The presence of CpG islands in the promoter region does not necessarily imply that these genes are highly likely to be epigenetically regulated. To confirm that, we investigated whether these genes are epigenetically regulated by analyzing the *in silico* dataset of lung epithelial cells (NHBE and A549) and monocyte cells (U937) treated with AZA. Out of the 19 genes, seven genes (*HSPA1L*, *ULBP2*, *ULBP3*, *KLRG2*, *DERL3*, *PRADC1*, and *FOXJ1*) genes were upregulated following AZA treatment in both epithelial cell lines confirming their epigenetic regulation (Figure 3C). Seven other genes were also upregulated in AZA treated monocyte cells, out of which two were shared with epithelial cells (*HSPA1L* and *ULBP2*). Supplementary Figure 2A shows the heat map correlation of epigenetic candidate gene expression in COVID-19 lung with AZA treated lung epithelial cell lines and immune-related cell line. We also examined the mRNA levels for different DNA methyltransferases (DNMT1, DNMT3A, and DNMT3B) in SARS-COV-2 infected epithelial cells (NHBE and A549). Interestingly, all three DNA methyltransferases were significantly downregulated following infection (Supplementary Figure 2B). However, similar inhibitions of DNMTs were not seen in COVID-19 patient’s lung tissues (data not shown).

### qMSP and RT-PCR Confirms *HSPAIL* Gene Promoter Hypomethylation With SARS-CoV-2 Infection

**FIGURE 4:**
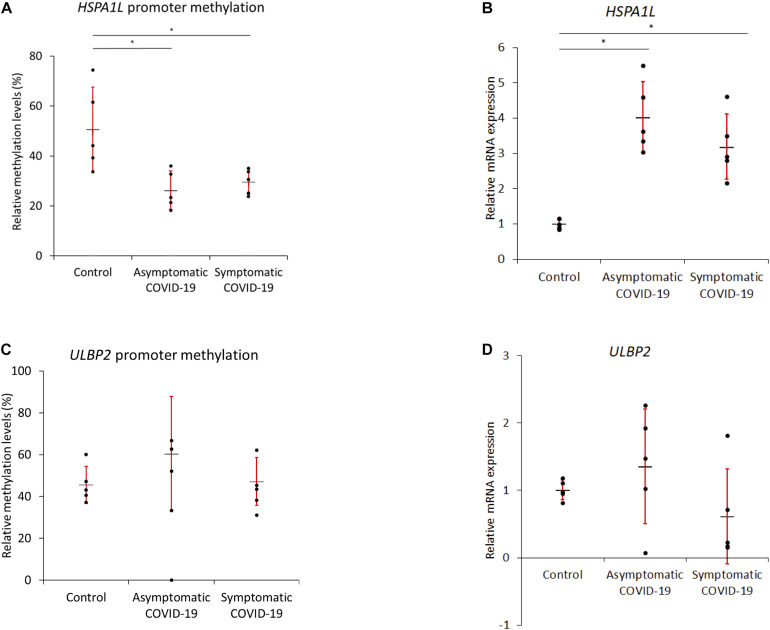
Quantitative Methylation-specific PCR and real-time PCR confirms *HSPAIL* promoter hypomethylation-overexpression with SARS-CoV-2 infection. Methylation levels of *HSPAIL* and *ULBP2* were measured by qMSP. **(A,B)** For the *HSPA1L* gene, the methylation levels were significantly lower but mRNA expression was significantly higher in both asymptomatic and symptomatic COVID-19 compared to healthy controls. **(C,D)** No difference in *ULBP2* methylation levels or mRNA expression was observed between the blood of healthy and COVID-19 samples. *P-value < 0.05 (*n* = 5 for each group); the red error bar shows standard error of mean (SEM) and the black horizontal bar shows the mean value.

Gene promoter methylation levels of *HSPAIL* and *ULBP2* were measured using qMSP. These two genes were upregulated in COVID19-lungs and SARS-CoV-2 infected lung epithelial cells and were the only genes upregulated upon AZA treatment in both lung epithelial cells as well as immune cells. For the *HSPA1L* gene, the promoter methylation level was significantly lower (hypomethylation) and the mRNA expression was significantly higher in both asymptomatic and symptomatic COVID-19 compared to healthy controls (Figures 4A,B). However, there was no significant difference in promoter methylation levels of *ULBP2* gene and the mRNA expression when comparing healthy individuals vs. COVID-19 patients (Figures 4C,D).

## Discussion

COVID-19 is associated with severe structural lung injury, hyper-inflammation, and disruption of the host immune system. The SARS-CoV-2 virus enters the body through the upper respiratory tract and accumulates at the target site of lung tissue triggering series of genetic and epigenetic modifications. Most of the previous research has focused on the genetic outcomes of the SARS-CoV-2 infection (Li et al., 2020; Ong et al., 2020; Saheb Sharif-Askari et al., 2020), whereas the epigenetic effects of this viral infection have been less described (Sawalha et al., 2020).

Epigenetic modifications include histone modification, DNA methylation, and gene ubiquitination. Previous studies have suggested that DNA methylation, rather than histone acetylation, is the main driver for the virus-induced epigenetic changes (Okamoto et al., 2014; Engdahl et al., 2017; Menachery et al., 2018). However, inconsistent DNA methylation patterns were reported in different viruses or even within the strains of the same virus. In general, viruses associated with cancers including the hepatitis B virus (HBV) and hepatitis C virus (HCV), were more likely to cause hypermethylation (Okamoto et al., 2014). In Influenza A (IVA) infection, the degree and nature of DNA methylation were different in H1N1 and H5N1 (Mukherjee et al., 2013; Menachery et al., 2018). IAV infection was showed to induce promoter hypomethylation in different inflammatory cytokine genes (Mukherjee et al., 2013). More recently, MHC gene loci were found to be hypermethylated with MERS-CoV and hypomethylated with SARS-CoV-1 (Menachery et al., 2018). Likewise, in our analyses of SARS-CoV-2 infection, out of 14 candidate epigenetic genes, 12 were upregulated suggesting hypomethylation, and only two genes were down-regulated which could be due to hypermethylation.

The virus-mediated change in methylation levels is attributed to the activity levels of different DNA methyltransferases (Macnab et al., 1988; Mukherjee et al., 2013; Menachery et al., 2018). It is then expected that viruses causing DNA demethylation to decrease the expression level of these enzymes. In our result, we have shown a significant decrease in levels of DNMT1, DNMT3A, and DNMT3B following SARS-CoV-2 *in vitro* infection (Supplementary Figure 1). However, similar effects on DNMTs were not seen in COVID-19 patient’s lung tissues, probably because those samples were obtained from dead patients after a severe disease and the virus-induced DNMT inhibition would be an early phase hallmark of infection.

Using multiple datasets, we have highlighted the genetic expression changes induced by severe COVID-19, which are most likely to be epigenetically modulated. *In silico* transcriptomic data of COVID-19 lung autopsies were used to identify the top differentially expressed genes in five individual cases compared to healthy control. Further, those genes were searched for CpG Islands in their promoter region. We confirmed this viral-induced gene upregulation using *in vitro* SARS-CoV-2 infected epithelial cells (NHBE and A549). Moreover, the epigenetic regulation of the selected genes was corroborated in AZA treated lung epithelial (NHBE and A549) cells as well as U937 immune cells. The genes that were upregulated following AZA treatment are highly likely to be epigenetically regulated *via* its promoter methylation. These epigenetic modifications could be the net outcome of one or multiple factors including the direct effect of different viral proteins, viral evasion mechanisms, host immune response, and infection driven inflammation and tissue injury.

Further, we suggest that these genes might be epigenetically modified either by the viral proteins or because of inflammation induced by a viral infection. The inflammatory immune response to SARS-CoV-2 involves an increased infiltration of neutrophils and monocytes to the infected lungs leading to a condition of chronic inflammation of the lung that involves a similar type of inflammation in the lungs of COPD patients. Hence, next we checked whether these genes are also upregulated in chronically inflamed COPD lung tissue. We found that these candidate genes were not expressed in COPD lungs indicating that these observed genetic changes are most likely mediated by SARS-CoV-2 induced infection and inflammation.

The main identified differentially expressed gene under DNA methylation epigenetic control had been previously associated with lung injury and remodeling (FOXJ1), viral protein synthesis, and replication (HSPA1L and DERL3) and immune system modulation (DERL3, KLRJ2, ULBP2, and ULBP3). The expression of *FOXJ1* was decreased in COVID-19 lung biopsies. SARS-CoV-2 could have induced the change partly by increasing the DNA methylation in the FOXJ1 promoter region, as seen in an upregulation of FOXJ1 in AZA treated cells. | However, the expression was not changed in SARS-CoV-2 infected lung epithelial cells.

*HSPA1L* and *ULBP2* were upregulated in COVID19-Lungs and SARS-CoV-2 infected lung epithelial cells and were the only genes upregulated upon AZA treatment in both lung epithelial cells as well as immune cells. To validate these results, we compared the promoter methylation levels of these two genes in the blood of healthy controls, asymptomatic, and severe COVID-19 patients. While there was no difference in methylation levels of *ULBP2*, methylation levels of *HSPA1L* were significantly decreased in both asymptomatic and severe COVID-19 blood samples. Moreover, mRNA expression levels were significantly higher in COVID-19 patients samples, both symptomatic and asymptomatic, vs. healthy control. This result could suggest that the HSPA1L gene is subjected to SARS-CoV-2 epigenetic regulation regardless of the severity of COVID-19 disease.

SARS-CoV-2-mediated epigenetic regulations are believed to facilitate the different stages of the viral life cycle from viral cell entry to replication and protein synthesis (Pruimboom, 2020). The level of *HSPA1L* expression was found to be significantly increased in COVID-19 lung biopsies and infected epithelial cells. *HSPA1L* gene, a heat shock protein 70 or Hsp70 protein, is involved in different biological processes including endocytosis, antigen processing and presentation, and protein processing in the endoplasmic reticulum (Lahaye et al., 2012). Hypomethylation and upregulation of this heat-shock protein could aid in viral protein synthesis and replication inside the host cell. More recently, Taguwa et al. (2019) demonstrated the functional role of different Hsp70 isoforms which is an essential protein required in host cell viral replication and pre- and post-entry events, and they also proposed an attractive use of Hsp70 inhibitors could control Zika viral replication and protein synthesis. The similar function of the *HSPA1L* gene could be attributed to COVID-19 lungs, suggesting that SARS-CoV-2 infected cells epigenetically upregulated the HSPA1L gene, hence Hsp70 proteins, to facilitate coronavirus replication in these host cells. Most of the available antiviral therapies are targeting viral proteins that are prone to mutations, whereas host targets, such as Hsp70 protein, have a broad spectrum of action and they are refractory to drug treatment (Taguwa et al., 2019).

Nowadays, the use of gene expression profiling data has been extensively employed for the identification of novel pathogenic pathways and therapeutic targets for several disorders including COVID-19 (Jeong et al., 2018; Cavalli et al., 2020; Li et al., 2020; Saheb Sharif-Askari et al., 2020). A recent study by Cavalli et al. (2020) has utilized the same COVID-19 lung autopsy RNAseq dataset, but their main aim was to profile the immune signatures. They only prioritized DEGs related to immune response by analyzing all the mean of all the infected samples vs. the negative control. However, in our study, we have considered each COVID-19 lung sample as individual and only technical replicate from one patient sample was compared against the negative control (pair-wise comparison). Later we filtered the commonly expressed genes in these five pair-wise comparisons. Then we investigated two of those genes for their likelihood to be epigenetic regulation upon SARS-CoV-2 infection using blood samples. We found that HSPA1L gene is hypomethylated and overexpressed in COVID-19 patients. We understand the limitation of our study that there is a possibility that the some of the transcriptional changes identified in the autopsy’s lungs of COVID-19 might reflect the changed cellular composition probably due to infiltration of the immune cells. Similarly, the hypomethylation in the HSPAIL gene could be due to a change in cellular composition as whole blood DNA methylation was analyzed. However, to rule out some of these effects, we confirmed that similar genetic changes were observed individually infected-lung epithelial cells and the immune cells treated with AZA.

In conclusion, for the first time, we have shown that compared to healthy individuals the *HSPA1L* was highly expressed in lung biopsies of patients with severe COVID-19 and lung epithelial cells infected with SARS-CoV-2. Also, the GTEx database analysis showed that HSPA1L gene expression is very low in normal lung tissues. Given that, *HSPA1L* gene promoter has CGI and AZA treatment of lung epithelial cells and immune cells showed differential modulation of *HSPA1L*, suggested the likelihood of epigenetic modulation of *HSPA1L* gene upon SARS-CoV-2 infection. We acknowledge that this analysis was obtained from a small cohort of patients as this was a pilot study. However, based on the *in silico* analysis and data from COVID-19 patients’ blood samples, we suggest that the *HSPA1L* could be considered as an epigenetic biomarker for diagnostic stratification of COVID19 patients in a much larger study and could be used as a potential target for to prevent the entry of SARS-CoV-2 virus into the host cells.

## Data Availability Statement

The original contributions presented in the study are included in the article/Supplementary Material, further inquiries can be directed to the corresponding author/s.

## Ethics Statement

The studies involving human participants were reviewed and approved by Dubai Scientific Research Ethics Committee (DSREC). The patients/participants provided their written informed consent to participate in this study.

## Author Contributions

JSM conceived the idea, designed, performed analysis, performed the experimental validation, prepared figures, drafted, and edited the manuscript. NS analyzed the part of the data and contributed in writing the manuscript. Z-GC and MH provided intellectual input and critically reviewed the manuscript for improvement. RH supervised the project and contributed to the idea, designing results, and manuscript writing. All authors reviewed and approved the final version of the manuscript for submission.

## Conflict of Interest

The authors declare that the research was conducted in the absence of any commercial or financial relationships that could be construed as a potential conflict of interest.

## Footnotes

1 https://www.ncbi.nlm.nih.gov/geo/

2 https://www.ncbi.nlm.nih.gov/geo/query/acc.cgi?acc=GSE150316

3 https://research.cchmc.org/pbge/lunggens/

4 https://www.gtexportal.org/home/

5 http://www.bioconductor.org/packages/release/bioc/html/limma.html

## Supplementary Material

Supplementary Figure 1 AZA treatments of epithelial and immune cells confirm the epigenetic regulation of 12 genes. **(A)** Heat map showing expression correlation of epigenetic candidate gene expression in COVID-19 lung plotted with AZA treated lung epithelial cell lines and immune-related cell line (vertical axis represents relative mRNA expression as fold change or in Log2FC). **(B)** Dataset for primary human lung epithelium (NHBE) mock-treated or infected with SARS-CoV-2 and transformed lung alveolar (A549) cells mock-treated or infected with SARS-CoV-2 was analyzed for relative mRNA expression of DNA methyltransferases (DNMT1, DNMT3A, and DNMT3B).

Supplementary Figure 2 Example of primary data showing the behavior and specificity of the designed primers on the methylation controls and unmethylated control and some of the tested samples.
