# Supplementary figures and images for: SARS-CoV-2 Infection-Induced Promoter Hypomethylation as an Epigenetic Modulator of Heat Shock Protein A1L (HSPA1L) Gene
Source: Front Genet. 2021 Feb 19;12:622271. doi: 10.3389/fgene.2021.622271 (PMC7933663; doi:10.3389/fgene.2021.622271)

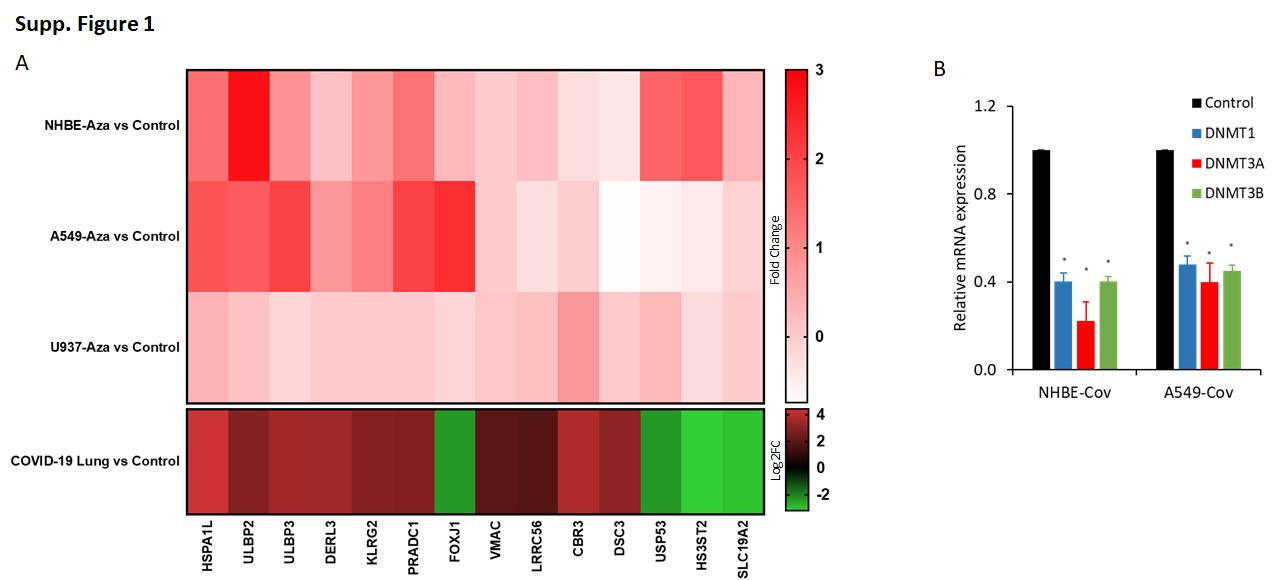

Supplement: Supplementary Figure 1 — AZA treatments of epithelial and immune cells confirm the epigenetic regulation of 12 genes. (A) Heat map showing expression correlation of epigenetic candidate gene expression in COVID-19 lung plotted with AZA treated lung epithelial cell lines and immune-related cell line (vertical axis represents relative mRNA expression as fold change or in Log2FC). (B) Dataset for primary human lung epithelium (NHBE) mock-treated or infected with SARS-CoV-2 and transformed lung alveolar (A549) cells mock-treated or infected with SARS-CoV-2 was analyzed for relative mRNA expression of DNA methyltransferases (DNMT1, DNMT3A, and DNMT3B). [file Image_1.tif]

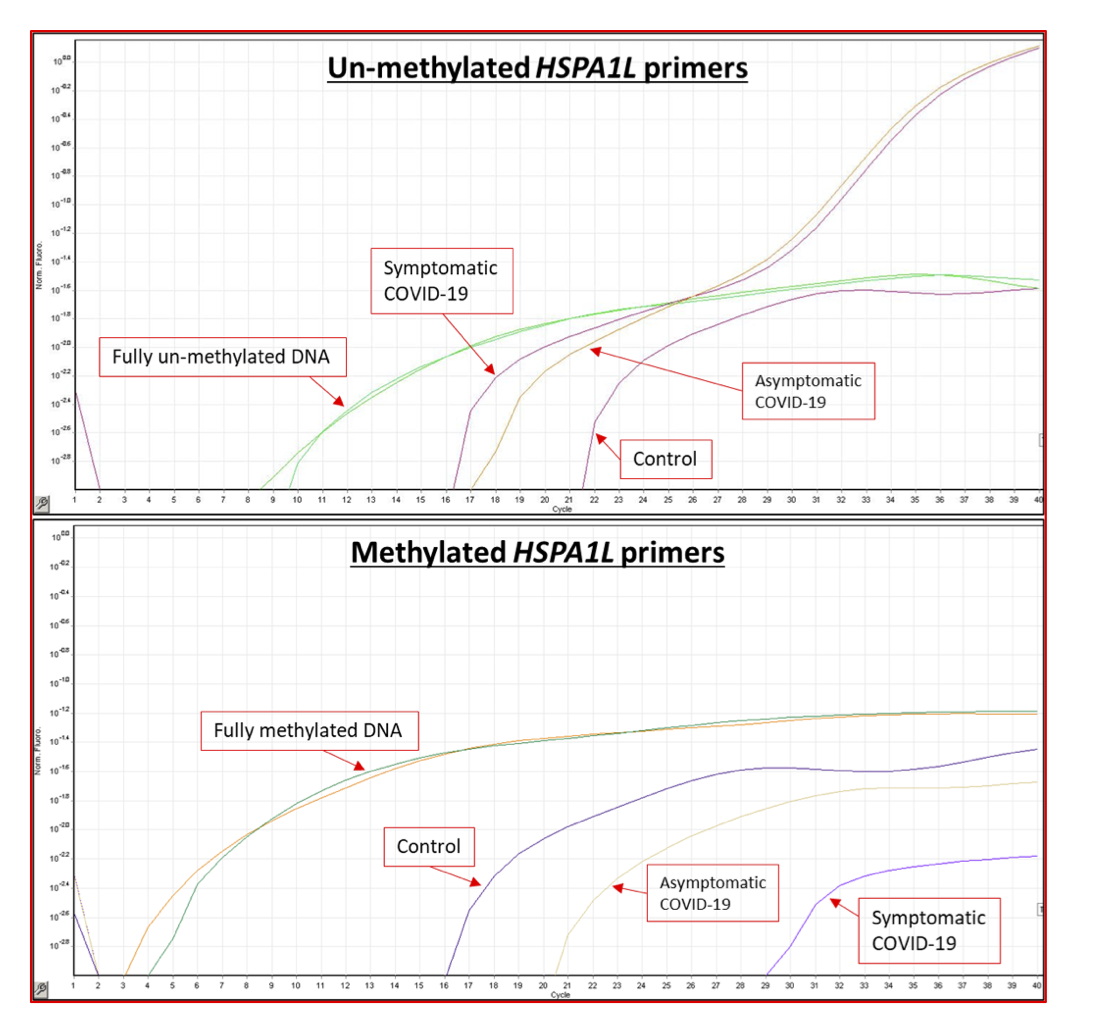

Supplement: Supplementary Figure 2 — Example of primary data showing the behavior and specificity of the designed primers on the methylation controls and unmethylated control and some of the tested samples. [file Image_2.tiff]
