# Supplementary material for: SARS-CoV-2 Infection-Induced Promoter Hypomethylation as an Epigenetic Modulator of Heat Shock Protein A1L (HSPA1L) Gene
Source: Front Genet. 2021 Feb 19;12:622271. doi: 10.3389/fgene.2021.622271 (PMC7933663; doi:10.3389/fgene.2021.622271)
Supplement: Supplementary file 3 [file Table_1.docx]

**Supplementary Table 1:** List and sequences for quantitative methylation specific PCR (qMSP) and quantitative real-time PCR primers (qRT-PCR).

| qMSP primers | | | | | |
| --- | --- | --- | --- | --- | --- |
| Gene | **F/R** | **M/U** | **Sequence (5' > 3')** | **Tm (^0^C)** | **Product (bp)** |
| *HSPA1L* | F  R | M | GTTTAATTGATAGGAAGGGTC  CATTATAACGTAACCGAACGATA | 52.2  56.1 | 157 |
| *HSPA1L* | F  R | U | GTTTAATTGATAGGAAGGGTT  CTCCATTATAACATAACCAAACAATA | 51.9  55.8 | 160 |
| *ULBP2* | F  R | M | GATAAGTTAGGGATTTCGCGG  GACTCCTACCAAAACACGATT | 59.9  55.8 | 179 |
| *ULBP2* | F  R | U | GATAAGTTAGGGATTTTGTGG  CCAACTCCTACCAAAACACAAT | 53.1  58.0 | 181 |
| qRT-PCR primers | | | | | |
| Gene | **F/R** | **Sequence (5' > 3')** | | **Tm (^0^C)** | **Product (bp)** |
| *HSPA1L* | F  R | TTACCGTGCCAGCCTATTTCA  AGCACATTAAGTCCAGCAATCA | | 61.5  60.0 | 79 |
| *ULBP2* | F  R | GTGGTGGACATACTTACAGAGC  CTGCCCATCGAAACTGAACTG | | 60.1  61.2 | 150 |
| *GAPDH* | F  R | CCAGGTGGTCTCCTCTGACTTC  ACATACCAGGAAATGAGCTTGACA | | 60.0  60.0 | 108 |
